# Spatial and seasonal dynamics of biogenic silica in a eutrophic marginal sea, the East China Sea

**DOI:** 10.1016/j.fmre.2023.07.005

**Published:** 2023-09-19

**Authors:** Yating Li, Xinyuan Zhang, Danna Wang, Xin Liu, Kuanbo Zhou, Bangqin Huang, Minhan Dai, Zhimian Cao

**Affiliations:** a State Key Laboratory of Marine Environmental Science, Xiamen University, Xiamen 361102, China; b College of Ocean and Earth Sciences, Xiamen University, Xiamen 361102, China; c College of the Environment and Ecology, Xiamen University, Xiamen 361102, China

**Keywords:** Biogenic silica, Spatial and seasonal variability, Diatom productivity, East China Sea, Marginal sea

## Abstract

Biogenic silica (BSi) is a key component of the marine silicon cycle, which is mainly driven by diatom metabolism notably contributing to primary production and export of particulate organic carbon (POC), particularly in ocean margins. Based on a large data set collected in the East China Sea (ECS), including BSi, fucoxanthin (Fuco), POC, and total suspended matter (TSM), we systematically explored the distribution and control of BSi in a typical eutrophic marginal sea. Spatially, BSi concentrations generally decreased from the shelf to the slope during all seasons, because the former is largely fed by river plumes and/or coastal currents enriched in nutrients favoring diatom growth. Abundant BSi was also observed in nearshore bottom waters probably influenced by sediment resuspension indicated by high TSM concentrations. Seasonally, BSi concentrations were on average higher in summer and autumn than in spring and winter, which reflects elevated diatom productivity during warm seasons. The BSi standing stock in the shallow water column of the ECS was significantly correlated with that of Fuco demonstrating diatoms’ dominant control on BSi dynamics. In addition, significant relationships between BSi and POC standing stocks were observed in summer and autumn, indicating the major role of diatoms in C fixation. By comparing with the northern South China Sea (NSCS), we suggested relatively small seasonal variability of BSi on the ECS shelf but significant decrease during cold seasons in the ECS slope. In the latter case, diatoms are ecologically and biogeochemically more important and more sensitive to the changing physical and/or chemical conditions than in the oligotrophic NSCS slope.

## Graphical abstract

**Figure:**
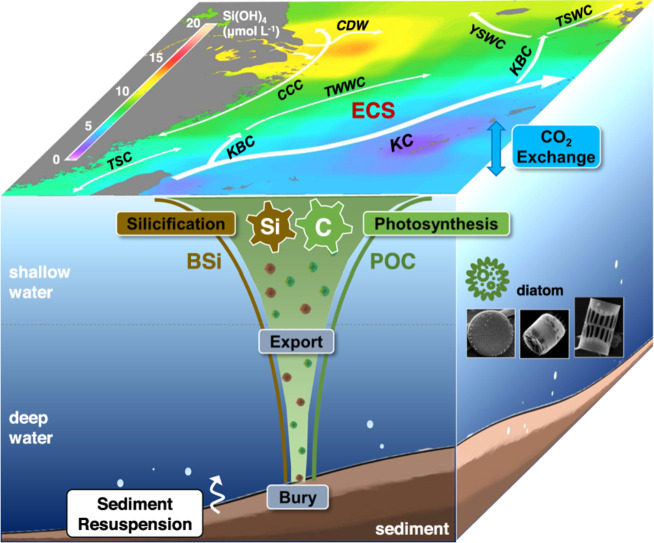

## 1 Introduction

Diatoms account for up to 40% of primary production (PP) and approximately 40% of particulate organic carbon (POC) export in the global ocean [1]. The biogeochemical cycle of silicon (Si) is thus of particular importance in the oceanic C cycle. In addition to photosynthesis, diatoms also take up dissolved silicate (Si(OH)_4_) from seawater to form a hard siliceous frustule, known as opal or biogenic silica (BSi). Consequently, BSi is widely used as a tool to reveal the spatio-temporal distribution of C production and export in the upper ocean [2]. While a number of studies aiming to understand BSi behaviors and its interaction with POC have been conducted in the open ocean [3], [4], [5], fewer BSi data have been obtained in ocean margins where diatom populations are more important under relatively eutrophic conditions [6,7].

The East China Sea (ECS) is the largest temperate marginal sea of the western North Pacific (wNP), which is characterized by a broad shelf largely influenced by the world's fourth largest river, the Changjiang (Yangtze River), and a direct exchange with the wNP via the western boundary current, the Kuroshio Current (KC). Rich supplies of nutrients from both the Changjiang discharge and upwelling of Kuroshio subsurface water stimulate high PP on the East China Sea shelf, leading to one of the most productive areas in the world's oceans [8], [9], [10]. The phytoplankton abundance is generally dominated by diatoms, which, however, show large spatial and seasonal variations. In spring, algal blooms with high biomass of diatoms (> 10^5^ cells L^−1^) are observed in the nearshore associated with the river plume. In summer/early autumn, the diatom biomass decreases rapidly from the inner shelf influenced by the plume water (∼10^5^ cells L^−1^) toward the shelf edge and slope influenced by the Kuroshio water (< 10^4^ cells L^−1^). In late autumn/winter, diatom abundances are overall the lowest (on average 10^3^ cells L^−1^) showing relatively uniform distributions over the ECS [11,12].

The changing roles played by diatoms in different regions and/or seasons in the ECS provide an opportunity to comprehensively examine BSi dynamics in a large marginal sea. However, data of BSi concentrations collected in the ECS water column [6,[13], [14], [15] are sparsely relative to those of BSi contents in the ECS sediments [14], [15], [16], [17], [18]. Therefore, mapping water column BSi distributions, along with relevant parameters such as fucoxanthin (Fuco, diatoms’ major marker pigment) and POC, is needed to develop a coherent picture of both Si and C cycling in the ECS. In this study, BSi in suspended particles was collected from the shelf to the slope in the ECS during all four seasons. Based on this field sampling, we systematically investigated the spatial and seasonal variability of BSi in the upper water column under various physico-biogeochemical conditions. Standing stocks of BSi were compared to those of Fuco, chlorophyll *a* (Chl-*a*), and POC, to explore the diatoms’ role in phytoplankton community and photosynthetic C fixation in a eutrophic marginal sea.

## 2 Materials and methods

### 2.1 Study area

**Fig. 1:**
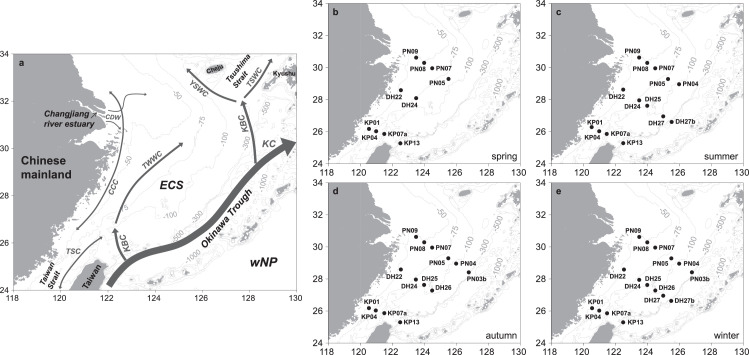
**Bathymetric map of (a) the East China Sea (ECS) showing the locations of sampling stations in (b) spring (May to June 2011), (c) summer (August 2009), (d) autumn (November to December 2010), and (e) winter (December 2009 to January 2010).** In (a), the surface circulation pattern is schematically shown according to ref. [19]. CDW: Changjiang Diluted Water; CCC: China Coastal Current; TSC: Taiwan Strait Current; TWWC: Taiwan Warm Current; YSWC: Yellow Sea Warm Current; TSWC: Tsushima Strait Warm Current; KBC: Kuroshio Branch Current; KC: Kuroshio Current; wNP: the western North Pacific. The CDW, the CCC, and the TSC generally flow northward during summer southwest monsoon season and southward during winter northeast monsoon season. All maps were generated based on the standard map GS(2020)4619.

The ECS, having an area of ∼0.7 × 10^6^ km^2^ and an average depth of ∼300 m, extends from Cheju Island in the north to the northern coast of Taiwan in the south, and is bounded by the Okinawa Trough in the east and the coast of China in the west (Fig. 1a). The main KC flows northeastward along the ECS continental slope, while the Kuroshio Branch Current (KBC) flows westward onto the shelf. The KBC west of Kyushu forms the origin of the Tsushima Strait Warm Current flowing into the Japan Sea and the Yellow Sea Warm Current flowing into the Yellow Sea. The KBC north of Taiwan forms the origin of the northward expanding Taiwan Warm Current, which is also fed by the Taiwan Strait Current (TSC) in summer (Fig. 1a; [19]). The East Asian Monsoon largely controls the pathway of the TSC, the China Coastal Current (CCC), and the Changjiang Diluted Water (CDW), which is generally towards the north and east during summer southwest monsoon season but southward during winter northeast monsoon season (Fig. 1a; [19]). Other fresh water inputs include plumes from the Ou River and the Min River, which disperse in the middle and south of the ECS, respectively.

### 2.2 Sampling and analyses

#### 2.2.1 Sampling

Sampling was conducted onboard the R/V *Dongfanghong* II during spring (May to June 2011), summer (August 2009), autumn (November to December 2010), and winter (December 2009 to January 2010) cruises to the ECS. Vertical profiles of BSi, Fuco, Chl-*a*, POC, and total suspended matter (TSM) were obtained in the upper 150 m of the water column, where diatom growth mainly occurs, at 10, 14, 14, and 16 stations along 3 transects (KP, DH, and PN in the south, middle, and north of the ECS) extending from the shelf to the slope during the spring, summer, autumn, and winter surveys, respectively (Fig. 1b-e; Table 1).

**Table 1:** **Temperature, salinity, silicate (Si(OH)**_**4**_**), biogenic silica (BSi), poticulate organic carbon (POC), and total suspended matter (TSM) concentrations in the upper 150 m of the water column at different stations in the East China Sea in various seasons.**

Station	Depth	Temperature	Salinity	Si(OH)_4_^a^	BSi	POC^b^	TSM
	(m)	(°C)		(µmol L^−1^)	(µmol L^−1^)	(µmol L^−1^)	(mg L^−1^)
*spring (May-June 2011)*							
KP01	0	22.75	34.24	3.00	0.76	11.29	0.35
26.2°N	15	22.72	34.22		0.89	14.43	0.49
120.6°E	25	22.74	34.24	2.97	0.90	8.69	0.37
	50	22.51	34.19	2.91	0.60	6.22	0.38
KP04	0	24.00	34.35	1.53	0.20	6.09	0.24
26.1°N	15	23.98	34.35		0.36	8.54	0.25
121.0°E	25	24.00	34.35	1.56	0.30	6.25	0.32
	50	23.44	34.36	2.51	0.27	4.44	0.28
	75	22.34	34.40	6.22	0.26	5.12	2.16
KP07a	0	23.31	34.29	3.19	0.26	6.66	0.38
25.9°N	15	23.31	34.29	3.11	0.67	6.31	0.35
121.5°E	25	23.27	34.29	3.09	0.45	5.57	0.26
	50	23.13	34.30	3.14	0.55	4.73	0.33
	75	21.94	34.35	6.59	0.81	6.44	2.30
KP13	0	25.51	33.97	2.25	0.30	5.76	0.23
25.3°N	25	24.52	34.03	2.38	0.61	5.95	0.24
122.5°E	50	23.32	34.21	3.76	0.58	4.99	0.29
	75	20.08	34.56	7.63	0.38	2.29	0.12
	100	18.84	34.60	8.60	0.13	2.01	0.12
	125	15.96	34.59		0.05	2.40	0.12
	150	14.96	34.56	15.88	0.04	1.31	0.12
DH22	0	20.88	32.69	7.30	1.72	16.24	0.72
28.6°N	25	19.82	33.27	7.55	0.93	6.95	0.43
122.5°E	50	18.49	34.37	12.62	0.13	9.27	4.60
DH24	0	23.41	34.24	1.68	0.01	6.61	0.23
28.1°N	25	22.85	34.21	1.66	0.05	6.17	0.16
123.5°E	50	22.58	34.15	4.12	0.06	4.58	0.16
	75	18.28	34.59	13.04	0.04	3.87	1.15
	85	18.28	34.59	13.01	0.05	4.33	1.06
PN09	0	20.79	32.09	8.60	0.06	14.02	0.15
30.7°N	15	20.76	32.06	8.62	0.03	9.07	0.40
123.5°E	25	20.01	32.82	16.59	0.02	6.40	0.28
	50	17.43	34.09	16.54	0.02	6.90	3.30
PN08	0	19.97	31.56	10.35	0.15	12.92	0.35
30.3°N	15	18.67	31.82	10.44	0.08	8.00	0.29
124.0°E	25	18.15	34.08	12.67	0.02	5.85	1.29
	50	18.12	34.09	12.71	0.01	6.85	1.43
PN07	0	21.61	33.02	5.15	0.05	14.46	0.27
30.0°N	15	21.72	33.29	3.25	0.05	8.87	0.21
124.5°E	25	20.99	34.06	12.77	0.19	6.26	1.29
	50	18.20	34.13	12.98	0.02	5.79	1.43
PN05	0	20.44	32.05	7.91	0.69	17.18	0.32
29.3°N	25	18.32	33.55	6.24	0.11	7.24	0.19
125.5°E	50	17.32	34.31	10.69	0.06	6.63	0.64
	75	17.32	34.31		0.04	4.61	0.64
	100	17.34	34.31	10.78	0.02	3.93	0.95
*summer (August 2009)*							
KP01	0	28.54	33.32	7.16	2.07	14.36	1.07
26.3°N	15	26.73	33.63	8.65	3.66	13.92	1.65
120.5°E	25	25.53	33.80	10.97	0.84	6.63	1.70
	50	23.94	33.95	9.73	1.18	8.95	7.95
KP04	0	28.65	33.45	7.38	0.77	7.56	0.66
26.1°N	15	27.59	33.38	8.89	1.66	11.98	1.48
121.0°E	25	26.69	33.63	5.50	0.84	6.06	0.80
	50	24.86	33.74	9.28	0.66	4.47	0.96
	75	23.33	34.08	11.07	0.91	10.04	8.28
KP07a	0	29.98	33.47	7.01	0.52	8.99	0.52
25.9°N	15	27.35	33.39	9.16	2.10	13.96	1.48
121.5°E	25	27.00	33.44	10.35	1.50	6.86	1.52
	50	24.56	33.76	8.33	0.45	4.91	2.82
	75	23.74	33.81	9.24	1.75	9.02	11.35
KP13	0	27.79	33.55	0.90	0.25	4.88	0.32
25.3°N	25	26.72	33.61	3.80	2.35	6.20	0.57
122.5°E	50	23.32	34.28	7.49	0.15	1.61	0.21
	75	20.03	34.58	8.30	0.04	0.69	0.12
	100	18.55	34.63	7.51	0.05	1.60	0.11
	125	17.24	34.60	11.04	0.13	0.54	0.11
	150	16.06	34.57	15.12	0.21	1.23	0.12
DH22	0	30.06	30.57	5.00	2.55	2.22	1.43
28.7°N	25	19.96	34.16	14.07	1.00	6.26	2.16
122.5°E	50	18.62	34.30	13.16	2.16	13.25	4.03
DH24	0	29.25	33.42	2.66	0.03	4.43	0.10
28.0°N	25	27.81	33.42	2.84	0.04	3.40	0.15
123.5°E	50	26.67	33.58	2.96	0.05	5.11	0.22
	75	24.66	33.85	10.41	0.09	4.03	0.82
	85	18.52	34.41	9.95	0.10	5.63	1.45
DH25	0	29.93	33.43	2.57	0.01	3.90	0.11
27.7°N	25	28.29	33.79	2.26	0.03	2.39	0.06
124.0°E	50	25.77	33.66	8.95	0.08	3.44	0.10
	75	18.68	34.44	13.62	0.17	2.48	0.68
	100	18.35	34.47	13.51	0.14	4.72	2.07
DH27	0	29.06	34.00	0.16	0.18	4.12	0.21
27.0°N	25	24.75	33.96	0.16	0.12	3.91	0.24
125.0°E	50	23.17	34.03	6.39	0.23	3.33	0.36
	75	20.20	34.62	8.61	0.18	2.25	0.25
	100	17.95	34.60	13.02	0.18	2.56	0.74
DH27b	0	28.61	33.75	1.18	0.03	3.13	0.13
26.7°N	25	27.20	34.11	1.09	0.13	2.89	0.09
125.5°E	50	25.40	34.37	0.93	0.24	5.33	0.28
	75	24.11	34.47	3.46	0.94	1.76	0.09
	100	22.82	34.60	4.57	0.13	1.19	0.07
	125	21.18	34.68	5.20	0.09	0.72	0.10
	150	18.98	34.67	11.44	0.14	1.20	0.08
PN09	0	28.83	32.21	0.96	0.52	11.99	1.21
30.7°N	15	26.68	33.67	4.34	0.10	7.71	0.18
123.5°E	25	24.31	33.95	9.30	0.14	8.38	0.25
	50	23.01	34.14	13.44	0.15	9.47	0.37
PN08	0	29.58	31.89	0.89	0.02	5.04	0.40
30.3°N	15	28.13	33.56	3.18	0.04	4.42	0.37
124.0°E	25	26.08	33.73	6.39	0.07	7.05	2.26
	50	25.75	33.82	21.22	0.89	10.16	7.43
PN07	0	29.62	33.65	2.13	0.01	2.59	0.11
30.0°N	15	29.42	33.62	2.18	0.02	2.92	0.09
124.5°E	25	28.46	33.57	2.31	0.03	3.17	0.28
	50	25.56	33.96	8.16	0.06	4.53	2.17
PN05	0	29.83	33.57	2.24	0.01	3.23	0.13
29.3°N	25	28.39	33.67	2.07	0.02	3.22	0.15
125.3°E	50	24.64	34.09	5.23	0.11	4.93	0.14
	75	21.56	34.36	13.49	0.04	4.01	1.74
	100	21.54	34.36	13.50	0.11	4.39	2.21
PN04	0	29.89	33.75	1.95	0.01	3.47	0.07
29.0°N	25	29.05	33.85	1.85	0.01	2.62	0.12
126.0°E	50	28.34	33.88	1.91	0.04	2.97	0.09
	75	24.22	34.16	5.39	0.05	3.04	0.27
	100	20.67	34.50	11.46	0.05	3.69	1.36
*autumn (November-December 2010)*							
KP01	0	21.08	33.88	7.54	0.35	5.15	3.46
26.2°N	15	21.08	33.88		0.82	5.84	3.51
120.5°E	25	21.09	33.88	7.54	0.65	6.66	4.17
	50	21.12	33.89	7.61	1.04		8.73
KP04	0	21.63	34.35	4.78	0.60	5.64	1.06
26.1°N	15	21.64	34.35		0.63	5.69	1.06
121.0°E	25	21.65	34.35	4.84	0.70	5.23	1.34
	50	21.66	34.35	5.56	0.68	5.26	1.22
	75	21.66	34.35	4.89	0.67		1.08
KP07a	0	21.17	34.44	5.81	1.03	4.65	0.31
25.9°N	15	21.17	34.44		0.73	4.16	0.27
121.5°E	25	21.08	34.43	7.56	0.46	4.61	0.29
	50	20.03	34.58		0.10	2.89	0.67
	75	20.04	34.58	8.90		4.26	2.27
KP13	0	25.78	34.44	1.24	0.21	3.37	0.06
25.3°N	25	25.79	34.44	1.24	0.29	3.19	0.08
122.5°E	50	25.80	34.44	1.24	0.20	2.89	0.11
	75	25.71	34.47	1.24	0.15	2.51	0.10
	100	23.79	34.50	1.24	0.08	2.11	0.07
	125	21.53	34.61	5.47	0.05	1.70	0.06
DH22	0	20.57	34.26	5.09	0.92	4.74	0.77
28.6°N	25	20.59	34.27	5.27	0.74	4.72	0.77
122.5°E	50	19.97	34.43	5.78	1.77		13.97
DH24	0	21.58	34.45	1.24	0.25	4.64	0.12
28.0°N	25	21.59	34.45	1.24	0.37	3.61	0.12
123.5°E	50	21.59	34.45	1.24	0.38	4.24	0.29
	75	20.24	34.50	12.53	0.27	4.10	0.80
DH25	0	21.35	34.37	1.24	0.44	3.43	0.15
27.7°N	25	21.36	34.37	1.24	0.40	3.60	0.14
124.0°E	50	21.15	34.32	1.24	0.49	3.24	0.15
	75	21.21	34.34	1.24	0.39	3.74	0.15
	100	19.72	34.47	12.83	0.51	3.99	0.62
DH26	0	21.47	34.40	1.24	0.19	3.73	0.17
27.3°N	25	21.48	34.40	1.24	0.17	3.72	0.13
124.5°E	50	21.45	34.40		0.13	3.46	0.12
	75	21.40	34.40	1.24	0.16	4.00	0.14
	100	18.91	34.58	13.77	0.23	3.17	0.37
PN09	0	17.34	31.92	15.79	4.09	18.39	0.82
30.7°N	15	17.51	32.07		3.78	17.81	0.99
123.5°E	25	18.68	33.17	12.84	1.20	9.69	1.15
	50	19.40	33.64	10.10	0.65	6.86	4.47
PN08	0	18.39	33.16	13.89	3.05	12.60	1.26
30.3°N	15	18.41	33.16		2.76	17.21	1.32
124.0°E	25	18.40	33.16	13.19	2.84	11.89	1.46
	50	18.76	33.42	11.98	2.29	13.51	4.82
PN07	0	18.85	34.01	5.93	0.64	9.20	1.14
30.0°N	15	18.86	34.01	5.95	0.63	6.22	1.02
124.5°E	25	18.85	34.01	5.98	0.47	5.68	0.79
	50	18.87	34.01	6.07	0.55	6.90	1.45
PN05	0	20.94	34.15	6.30	0.20	4.84	0.87
29.3°N	25	20.95	34.15	5.66	0.19	4.85	0.84
125.5°E	50	20.96	34.15	5.61	0.18	4.37	0.92
	75	20.96	34.15		0.26	5.04	1.44
	95	20.96	34.15	5.60	0.25	5.34	1.90
PN04	0	21.81	34.42	1.24	0.12	4.52	0.09
29.0°N	25	21.79	34.42	1.24	0.13	4.90	0.09
126.0°E	50	21.78	34.42	1.24	0.12	3.37	0.06
	75	21.35	34.43	10.93	0.13	2.88	0.09
	100	19.48	34.52		0.22	3.47	0.80
	115	19.47	34.51	15.51	0.30	4.16	1.39
PN03b	0	24.15	34.55	1.24	0.18	3.40	0.12
28.5°N	25	24.13	34.55		0.20	3.98	0.12
126.8°E	50	23.79	34.53	1.24	0.19	3.15	0.26
	75	23.52	34.53	1.24	0.16	2.54	0.11
	100	22.87	34.54	1.24	0.14	1.98	0.09
	125	21.42	34.58	5.53	0.13	0.96	0.07
	150	19.57	34.60	10.51	0.04	2.68	0.16
*winter (December 2009 to January 2010)*							
KP01	0	15.00	31.25	21.69	0.45	6.70	1.58
26.2°N	15	15.36	31.81	19.78	0.41	1.73	2.34
120.6°E	25	15.61	31.93	19.01	0.49	2.23	2.98
	50	16.80	32.52	14.89	0.86	1.76	13.43
KP04	0	18.50	33.16	8.75	0.48	5.79	2.63
26.1°N	15	18.28	33.16		0.49	8.31	3.42
121.0°E	25	18.29	33.16	8.67	0.47	7.17	4.95
	50	18.30	33.16	8.69	0.67	5.56	6.48
	75	18.29	33.15	8.75	0.85	9.35	8.51
KP07a	0	18.83	33.73	6.72	0.84	5.20	2.66
25.9°N	15	18.81	33.74	6.73	0.89	6.24	3.19
121.5°E	25	18.81	33.75	7.47	0.85	6.17	2.98
	50	18.70	34.12	7.52	1.42	7.61	10.07
	75	18.66	34.15		2.33	12.45	20.89
KP13	0	22.34	34.48	2.78	0.34	3.07	0.19
25.3°N	25	22.04	34.48	4.92	0.26	3.56	0.21
122.5°E	50	20.49	34.46	6.58	0.23	2.36	0.21
	75	19.19	34.44		0.13	1.88	0.19
	100	19.11	34.46	8.58	0.13	1.80	0.19
	125	18.73	34.52		0.12	1.61	0.18
	150	17.97	34.55	11.65	0.10	1.06	0.20
DH22	0	16.49	33.82	8.84	0.77	7.06	4.03
28.6°N	25	16.47	33.81	8.84	0.80	6.67	3.81
122.5°E	55	16.53	33.82	8.83	0.82	8.65	6.44
DH24	0	18.32	34.33	5.78	0.33	4.22	0.55
28.0°N	25	18.33	34.33	5.71	0.33	3.96	0.51
123.5°E	50	18.35	34.33	5.73	0.36	4.19	0.62
	75	18.36	34.33		0.37	3.77	0.75
	85	18.35	34.32	5.82	0.43	4.27	0.54
DH25	0	19.11	34.57	4.66	0.25	1.44	0.62
27.7°N	25	19.12	34.57	4.62	0.27	0.88	0.82
124.0°E	50	19.13	34.57	4.63	0.20	2.55	0.67
	75	19.14	34.57		0.25	0.78	0.69
	95	19.13	34.56	4.62	0.18	3.80	0.66
DH26	0	18.91	34.45	3.99	0.65	4.26	0.22
27.3°N	25	18.94	34.46	3.97	0.72	3.96	0.24
124.5°E	50	19.23	34.51	6.07	0.47	3.22	0.21
	75	19.23	34.52		0.71	3.51	0.19
	96	18.98	34.47	6.44	0.77	3.46	0.20
DH27	0	18.65	34.36	4.55	0.46	3.32	0.16
27.0°N	25	18.66	34.36	4.51	0.50	3.22	0.15
125.0°E	50	18.67	34.36	4.48	0.57	3.09	0.17
	75	18.67	34.36		0.58	3.07	0.16
	90	18.66	34.36	5.78	0.76	2.41	0.22
DH27b	0	23.19	34.57	0.60	0.07	1.50	0.22
26.7°N	25	23.23	34.59	0.60	0.07	1.95	0.19
125.5°E	50	23.28	34.61	0.60	0.06	2.70	0.11
	75	23.28	34.64	0.60	0.05	1.43	0.09
	100	22.35	34.58	0.93	0.12	1.74	0.08
	125	22.31	34.58	1.22	0.09	1.31	0.08
	150	21.77	34.73	1.13	0.07	1.12	0.09
PN09	0	16.19	33.98	7.74	0.59	5.35	1.78
30.7°N	15	16.19	33.98		0.53	5.28	1.74
123.5°E	25	16.20	33.98	7.76	0.46	5.51	1.85
	50	16.19	33.98	7.82	0.54	5.08	2.09
PN08	0	16.10	33.99	6.00	0.55	5.91	1.46
30.3°N	15	16.11	33.99		0.67	5.36	1.90
124.0°E	25	16.11	33.99	6.00	0.54	5.49	1.78
	48	16.11	33.98	5.99	0.50	5.40	1.94
PN07	0	17.35	34.04	7.74	0.28	3.79	0.63
30.0°N	15	17.36	34.04		0.29	3.84	0.67
124.5°E	25	17.37	34.04	7.71	0.29	3.62	0.69
	50	17.26	34.04	7.28	0.35	3.60	0.74
PN05	0	18.83	34.02	6.06	0.07	3.44	0.60
29.3°N	25	18.39	34.02	6.09	0.04	3.52	0.85
125.5°E	50	19.39	34.02		0.09	3.84	0.62
	75	18.84	34.02	6.05	0.09	3.56	0.59
	90	18.85	34.02	6.06	0.01	3.06	0.50
PN04	0	19.75	34.33	5.67	0.22	3.35	0.36
29.0°N	25	19.67	34.30	5.66	0.21	3.33	0.33
126.0°E	50	19.72	34.31	5.64	0.30	3.42	0.36
	75	19.64	34.27	5.62	0.24	3.12	0.32
	100	19.80	34.34	5.51	0.34	3.41	0.34
	115	19.80	34.33	5.49	0.04	3.61	0.27
PN03b	0	22.90	34.62	0.60	0.08	2.75	0.12
28.5°N	25	23.90	34.62		0.08	2.38	0.10
126.8°E	50	22.77	34.61	0.60	0.08	2.16	0.14
	75	20.87	34.54	1.05	0.29	2.71	0.16

a The limit of quantificaiton for Si(OH)_4_ is 0.40, 0.16, 1.24, and 0.60 µmol L^−1^ during measurement of samples collected in spring, summer, autumn, and winter, respectively. Si(OH)_4_ data derived from refs. [11,12].

b POC data derived from ref. [23].

Seawater samples were collected with Niskin bottles attached to a rosette sampler. Samples for BSi and TSM analyses were obtained by filtering 2–4 L of seawater through 47 mm diameter Millipore polycarbonate membrane filters (0.4 µm pore size), which were dried overnight onboard at 50 °C and stored in polycarbonate dishes for analysis on land. Samples for Fuco and Chl-*a* analyses were obtained by filtering 4–6 L of seawater through 47 mm diameter Whatman glass fiber GF/F filters (0.7 µm pore size), which were folded and stored in liquid nitrogen until analysis. 4–8 L of seawater was filtered through 25 mm diameter Whatman QM-A quartz microfiber filters (1.0 µm pore size) to determine POC concentrations. Samples for nutrient analyses were obtained by filtering ∼250 mL of seawater through 47 mm diameter nitrocellulose acetate filters (0.45 µm pore size) into acid pre-cleaned polyethylene bottles.

#### 2.2.2 Analysis of BSi

BSi deposited on the membrane filters was analyzed using a two-step, wet-alkaline digestion method [20]. Filters were subjected to digestions using 4 mL of 0.2 M NaOH at 100 °C for 40 min twice. Concentrations of Si in solution of both digestions were determined using a Technicon AA3 Auto-Analyzer (Bran+Luebbe GmbH) with a precision of ±2% (1 standard deviation, 1SD). To correct for the lithogenic silica (LSi) fraction during the first digestion, aluminum (Al) concentrations were measured using an Agilent 7700x quadrupole-Inductively Coupled Plasma-Mass Spectrometer with a precision of ±1% (1SD). BSi concentrations were thus calculated:

(1) $$\left[\text{BSi}\right]={\left[\text{Si}\right]}_{1}-{\left[\text{Al}\right]}_{1}\times {\left(Si/Al\right)}_{2}$$

where [Si]_1_ and [Al]_1_ denote Si and Al concentrations, respectively, in the first digest solution; (Si/Al)_2_ represents the ratio obtained in the second digest solution, which is characteristic of the silicate minerals present in the sample [20]. LSi fraction in the first digest solution ranged between < 10% and > 60% generally decreasing from nearshore to offshore samples. Since the interference introduced by LSi was not negligible and showed spatial and seasonal variations, the second digestion is indeed required for water column BSi measurements in marginal seas receiving large terrestrial inputs.

Using this method we analyzed the BSi data along with two sediment reference materials: Still Pond and Lewis Lake. The measured SiO_2_ values (mean %wt. ± 1SD, *n* = 3: Still Pond = 2.78 ± 0.46 and Lewis Lake = 44.8 ± 1.12) well agree with those obtained during a previous interlaboratory comparison (Still Pond = 2.82 ± 1.17 and Lewis Lake = 44.3 ± 9.38) [21]. Repeated measurements of selected filter samples yielded an uncertainty for the entire procedure of < ±10% (1SD, *n* = 10).

#### 2.2.3 Analyses of other parameters

Vertical profiles of seawater temperature and salinity were determined using a calibrated SBE-19-plus Conductivity-Temperature-Depth (CTD) recorder (Sea-Bird) attached to the rosette sampler. Nutrients were analyzed onboard using a Technicon AA3 Auto-Analyzer. The precisions for nitrate+nitrite (NO_x_), phosphate (PO_4_), and Si(OH)_4_ were ±1%, ±2%, and ±3%, respectively (1SD; [22]). Before BSi analysis, TSM was determined by drying at 50 °C and weighing the membrane filters used for filtering. After carbonate removal by concentrated HCl acid fumigation for 24 h at room temperature, POC was analyzed using a PE-2400 SERIES II CHNS/O analyzer with a precision of < ±10% (1SD). Fuco and Chl-*a* were analyzed by extraction with 2 mL of a N, N-dimethylformamide solution and measurement of extracts using an Agilent series 1100 High Performance Liquid Chromatograph [12]. Nutrient, Fuco, and Chl-*a* data were published by refs. [11,12], while POC data were published by ref. [23].

### 2.3 Estimate of BSi standing stock

At each station, the BSi standing stock in the shallow water column was calculated by integrating BSi concentrations from the surface to a selected depth, which is between the layer of subsurface Chl-*a* maximum and the zone significantly influced by sediment resuspension indicated by extremely high TSM concentrations. For a given station, a same integrating depth was applied for data collected during all four seasons. Integrations were performed manually using a trapezoidal approximation, which was also used to calcuate standing stocks of Fuco, Chl-*a*, and POC (Table 2).

**Table 2:** **Standing stock of biogenic silica (BSi), fucoxanthin (Fuco), chlorophyll*****a*****(Chl-*****a*****), and particulate organic carbon (POC) in the shallow water column of the East China Sea at different stations in various seasons**.

Station	Bottom	Integrating depth	BSi standing stock	Fuco standing stocka	Chl-*a* standing stocka	POC standing stockb
	(m)	(m)	(mmol m^−2^)	(mg m^−2^)	(mg m^−2^)	(mmol m^−2^)
*spring (May-June 2011)*						
KP01	66	25	21.40	3.87	25.41	308.54
KP04	81	25	7.46	1.34	19.69	183.61
KP07a	76	25	12.65	1.86	17.84	156.68
KP13	630	100	44.53	7.47	57.39	427.65
DH22	63	25	33.17	7.63	37.87	289.80
DH24	86	50	2.15	0.97	28.23	294.24
PN09	57	25	0.99	1.53	18.98	250.51
PN08	52	15	1.72	1.00	14.18	156.89
PN07	66	25	1.94	1.94	24.54	250.58
PN05	102	50	12.15	4.14	36.61	478.59
*summer (August 2009)*						
KP01	59	25	65.50	9.64	29.83	314.89
KP04	80	25	30.69	4.13	15.10	236.80
KP07a	77	25	37.61	6.74	20.42	276.22
KP13	574	100	67.51	9.11	28.06	293.59
DH22	58	25	44.35	6.32	16.14	105.88
DH24	88	50	1.99	0.89	20.56	204.08
DH25	97	50	1.73	0.76	15.93	151.41
DH27	117	50	8.07	1.92	15.55	190.85
DH27b	1061	100	34.95	10.31	42.03	303.45
PN09	59	25	5.82	1.86	15.37	228.21
PN08	51	15	0.42	0.17	5.28	70.94
PN07	66	25	0.40	0.11	6.13	71.75
PN05	94	50	2.04	0.65	20.63	182.50
PN04	114	50	0.98	0.22	13.40	145.94
*autumn (November-December 2010)*						
KP01	65	25	16.12	2.47	10.57	144.87
KP04	82	25	15.89	2.39	13.59	139.64
KP07a	76	25	19.20	4.22	24.04	109.95
KP13	559	100	19.86	4.10	53.05	283.07
DH22	64	25	20.64	2.55	14.29	118.29
DH24	85	50	17.11	1.96	26.89	201.23
DH25	98	50	21.61	2.30	24.75	173.44
DH26	101	50	8.43	1.59	20.21	182.83
PN09	59	25	83.95	21.89	53.37	408.93
PN08	53	15	43.58	9.98	25.50	223.53
PN07	67	25	15.09	3.69	16.08	175.14
PN05	97	50	9.53	2.83	18.57	236.28
PN04	119	50	6.15	1.50	24.50	221.25
PN03b	208	50	9.55	2.44	29.17	181.40
*winter (December 2009 to January 2010)*						
KP01	64.4	25	10.92	0.82	9.26	83.06
KP04	84	25	12.09	1.42	9.47	183.11
KP07a	78	25	21.74	1.80	10.48	147.91
KP13	570	100	21.27	4.68	52.58	255.69
DH22	61	25	19.55	1.55	9.65	171.55
DH24	87	50	16.91	3.61	29.87	204.19
DH25	99	50	12.34	2.45	23.42	71.91
DH26	100	50	32.07	3.88	27.45	192.55
DH27	117	50	25.26	3.04	26.43	160.46
DH27b	1269	100	6.78	1.17	31.65	192.59
PN09	59	25	13.28	2.24	12.96	133.65
PN08	50	15	9.12	1.32	6.78	84.53
PN07	67	25	7.23	1.66	12.23	94.51
PN05	92	50	2.88	1.29	15.18	178.78
PN04	118	50	11.80	2.48	23.69	167.94
PN03b	208	50	3.98	0.76	25.94	120.75

^a^ Fuco and Chl-*a* data derived from ref. [12]. ^b^ POC data derived from ref. [23].

## 3 Results

### 3.1 Hydrography

**Fig. 2:**
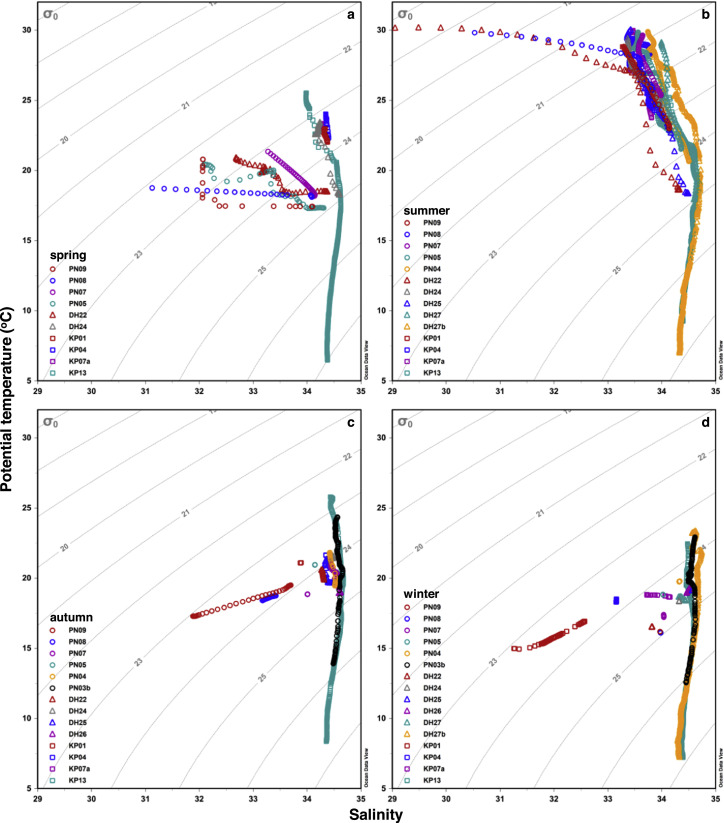
**Potential temperature versus salinity (T-S diagrams) in the upper 500m of the water column of the East China Sea in (a) spring, (b) summer, (c) autumn, and (d) winter at various study locations.** Gray lines indicate the isopycnals (σ_0_). The plot was generated with Ocean Data View [38].

As demonstrated by the temperature-salinity (T-S) diagrams, the hydrographic properties of ECS waters showed distinct spatial and seasonal variations in the upper 500 m of the water column (Fig. 2).

In spring, surface potential temperature (θ) and salinity varied within a range of 18.8–25.5 °C and of 31.1–34.4, respectively (Fig. 2a). The lowest value of both parameters in surface waters was observed at station PN08, which marks admixture of a cold and fresh water mass that might be the CCC and/or the CDW. The surface salinity at station PN07 was > 33.0 but < 34.0 and gradually increased with increasing water depth or density (σ_0_) levels along with decreasing θ, which represents a typical T-S distribution pattern of ECS shelf waters without either inputs of fresh waters or intrusion of the KBC. Waters at stations PN09, PN05, and DH22 were most likely mixture of those at stations PN08 and PN07. High surface salinities of > 34.0 were observed at stations along transect KP and station DH24, mainly resulting from exchange with the saline Pacific water. A subsurface salinity maximum of > 34.6 was observed around 100 m (σ_0_ ∼25.0) at the slope station KP13 (Fig. 2a), which is an imprint of the North Pacific Tropical Water.

In summer, surface θ of 28.8–30.1 °C was the highest during all four seasons, while surface salinity varied within a relatively wide range of 29.0 to 34.0 (Fig. 2b). The surface waters (σ_0_ < 17.5) at station DH22, with θ > 30.0 °C and salinity < 29.5, featured river plumes most likely sourced from the Ou River. The salinity of the surface waters (σ_0_ ∼18.5) at station PN08 remained low at < 31.0 while θ was > 29.5 °C, reflecting weakened influence of river plumes including the CDW. T-S distribution patterns at other shelf stations were overall comparable to those of the upper 75–100 m (σ_0_ < 24.5) at slope stations DH27b and KP13, which are characterized by stratification showing increasing salinity and decreasing θ with increasing water depth. Their surface salinities varied between 33.3 and 34.0 reflecting typical ECS waters with minor influence of either river plumes or the KBC. Due to the latter, the subsurface salinity maximum of > 34.7 was distinct around 140 and 95 m (σ_0_ ∼24.7) at stations DH27b and KP13 (Fig. 2b).

In autumn, surface θ and salinity varied within a range of 17.3.0–25.8 °C and of 31.9–34.6, respectively (Fig. 2c). Stations PN09 and PN08 generally showed an increase trend of both θ and salinity from the surface to the bottom, mainly resulting from the occupation or influence of the cold and fresh CCC in the surface. Other sampling areas having salinities > 33.9 were not affected by any fresh water inputs, among which stations PN07, PN05, KP01, and KP04 showed completely invariable θ and salinity due to thorough mixing of the entire water column. However, stations along transect DH, as well as stations PN04 and KP07, were slightly stratified, displaying slow increase in salinity and decrease in θ with increasing σ_0_ levels. At stations PN03b and KP13 in the slope, the subsurface salinity maximum of 34.7 around 150 m (σ_0_ ∼24.5) was slightly higher than the corresponding surface salinity of 34.5 (Fig. 2c), suggesting enhanced intrusion of the KBC and vertical mixing in the ECS during autumn.

In winter, surface θ of 15.0–23.3 °C was overall lower than in other three seasons, while surface salinity varied within a range of 31.3 to 34.6 (Fig. 2d). The innermost station KP01 was notably influenced by the CCC showing the lowest surface θ and salinity, both of which increased towards the bottom. In contrast, the entire water column at other stations on the ECS shelf was well mixed as demonstrated by nearly uniform θ and salinity values, in particular along transects PN and DH. The generally high salinity of > 33.8 indicates that waters in these sampling areas were dominated by saline waters including the KBC extending from the slope to the shelf, except that station KP04 was to some extent diluted by the KP01 water with lower salinities around 33.2. At stations PN03b and DH27b in the ECS slope, the subsurface salinity maximum was not clearly distinguishable owing to the comparable or even higher surface salinities above 34.6 (Fig. 2d), pointing to the strongest intrusion of the KBC during winter.

### 3.2 Biogenic silica concentrations

#### 3.2.1 Surface distribution

**Fig. 3:**
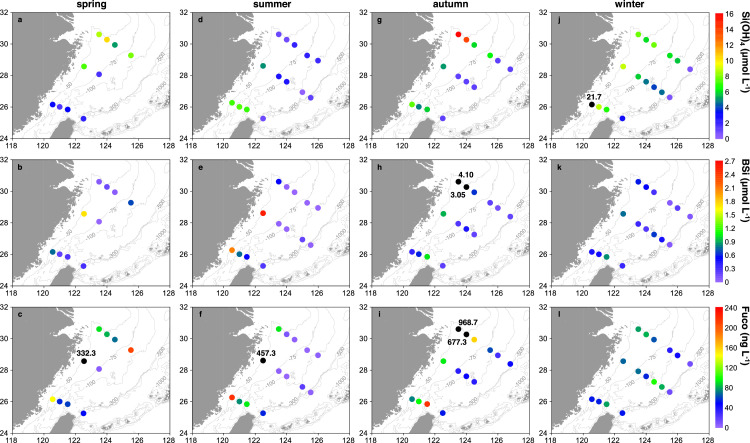
**Surface distributions of silicate (Si(OH)_4_), biogenic silica (BSi), and fucoxanthin (Fuco) concentrations in the East China Sea in different seasons.** (a-c) spring; (d-f) summer; (g-i) autumn; (j-l) winter. Numbers next to the solid black circles denote values beyond the set range of a given parameter in the plot.

Surface BSi concentrations in the ECS generally decreased from the shelf to the slope and were on average higher in summer and autumn than in winter and spring (Fig. 3; Table 1), showing spatial and seasonal distribution patterns, overall, similar to those obtained by ref. [6].

In spring, the highest BSi concentration of 1.72 µmol L^−1^ was observed in surface waters at station DH22 corresponding to extremely high Fuco concentratons of 332.3 ng L^−1^, whereas surface Si(OH)_4_ concentrations were in the middle of the range for the entire research domain (Fig. 3a-c). Along transect KP, both BSi and Fuco in surface waters overall decreased with increasing distance from the coast. Along transect PN, however, the outermost station PN05 displayed the highest surface BSi of 0.69 µmol L^−1^ and Fuco of 208.2 ng L^−1^ (Fig. 3b, c).

In summer, the highest concentration of BSi (2.55 µmol L^−1^) and Fuco (457.3 ng L^−1^) was also observed in surface waters at station DH22, which was fed by river plumes (Fig. 2b) stimulating high diatom productivity. Surface Si(OH)_4_, BSi, and Fuco concentrations along transect DH rapidly decreased from this innermost station to the offshore area (Fig. 3d-f). Si(OH)_4_ concentrations in surface waters along transect KP were overall higher than those along trasect PN. Corresponding to such different nutrient conditions, surface BSi and Fuco were also more abundant along transect KP than along transect PN (Fig. 3d-f).

In autumn, surface waters at stations PN09 and PN08 showed the highest concentration of Si(OH)_4_ (15.8 and 13.9 µmol L^−1^), BSi (4.10 and 3.05 µmol L^−1^), and Fuco (968.7 and 677.3 ng L^−1^) during all four seasons (Fig. 3g-i), reflecting extremely high diatom production in the CCC (Fig. 2c) enriched in nutrients. Along transect DH, surface Si(OH)_4_, BSi, and Fuco were notably higher at the innermost statoin DH22 than at other offshore stations, whereas station KP07a located in the middle of transect KP displayed the highest surface BSi of 1.03 µmol L^−1^ and Fuco of 216.9 ng L^−1^ (Fig. 3h, i).

In winter, the highest surface Si(OH)_4_ of 21.7 µmol L^−1^ during all four seasons was observed at station KP01 occupied by the CCC, which, however, did not greatly increase diatom growth showing intermediate BSi and Fuco concentrations (Fig. 3j-l). Stations KP07a and DH22 displayed enhanced BSi (around 0.80 µmol L^−1^) in surface waters corresponding to overall abundant Fuco and replete Si(OH)_4_. The extremely low surface BSi (0.07 and 0.08 µmol L^−1^) and Fuco (11.7 and 13.3 ng L^−1^) were observed at slope stations PN03b and DH27b under the condition of nearly depleted Si(OH)_4_ (Fig. 3j-l).

#### 3.2.2 Vertical distribution

**Fig. 4:**
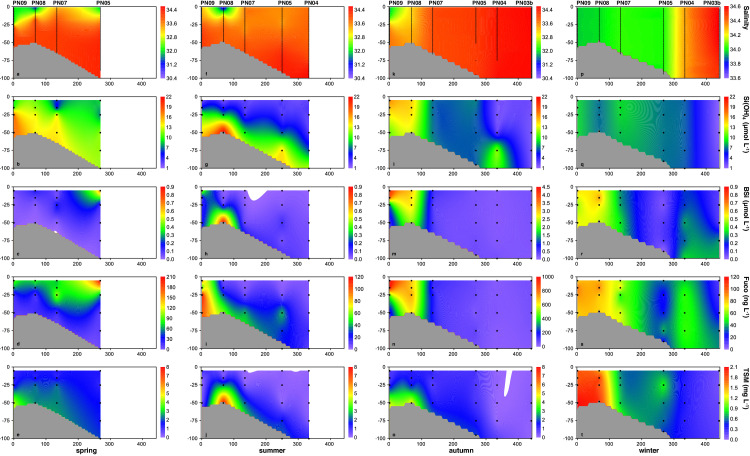
**Vertical distributions of salinity, silicate (Si(OH)_4_), biogenic silica (BSi), fucoxanthin (Fuco), and total suspended matter (TSM) concentrations above 100m along transect PN in the East China Sea (**Fig. 1**) in different seasons.** (a-e) spring; (f-j) summer; (k-o) autumn; (p-t) winter. Note that the scales for salinity in winter, BSi in autumn, Fuco in spring and autumn, and TSM in winter differ from those for a conresponding parameter in other seasons.

We select transect PN to show the vertical distribution of BSi, in addition to salinity, Si(OH)_4_, Fuco, and TSM, in the upper 100 m of the ECS. BSi concentrations were generally higher in the nearshore than the offshore area, except for during spring. The highest BSi concentration of > 4.0 µmol L^−1^ was observed in surface waters at the innermost station PN09 in autumn, which was approximately 4 to 6 times higher than the BSi maximum of 0.7–0.9 µmol L^−1^ for the other three seasons (Fig. 4; Table 1).

In spring, BSi and Fuco concentrations above 15 m at each station were overall higher than those in waters below, while peak values (0.69 µmol L^−1^ and 208.3 ng L^−1^) were observed in surface waters at statoin PN05. One exception is the 25 m water at station PN07 showing a subsurface maximum of both BSi and Fuco (0.19 µmol L^−1^ and 104.1 ng L^−1^), whereas its overlying waters had lower concentrations of Si(OH)_4_, BSi and Fuco (Fig. 4b-d). The surface BSi maxima at stations PN09, PN08, and PN05 mainly resulted from enhanced diatom production supported by relatively enriched Si(OH)_4_ in buoyant fresh waters (Fig. 4a, b). The subsurface BSi maximum at station PN07 corresponded to high values of both Si(OH)_4_ and TSM (12.8 µmol L^−1^ and 1.3 mg L^−1^). We contend that this maximum was most likely ascribed to diatom production rather than sediment resuspension, given that similar TSM levels in neighboring bottom/subsurface waters at stations PN08 and PN09 did not notably increase BSi and Fuco concentrations (Fig. 4b-e).

In summer, water column stratification was reflected by consistently increasing salinity and Si(OH)_4_ concentrations from the surface to the bottom or 100 m, while surface waters at station PN08 showed the lowest salinity of 31.9 and Si(OH)_4_ of 0.8 µmol L^−1^ (Fig. 4f, g). In this buoyant fresh water, however, both BSi and Fuco concentrations were also very low likely suggesting a post-bloom condition. The most abundant BSi (0.89 µmol L^−1^) was observed in bottom waters at station PN08, co-occurring with the highest TSM concentration of 7.4 mg L^−1^ (Fig. 4h-j). In this case, sediment resuspension probably played an important role. Nearby bottom waters at station PN09 showed the highest concentration of Fuco (around 100 ng L^−1^), which was overll abundant throughout the entire water column. Diatom production should be more important at this innermost station showing a surface maximum of BSi (0.52 µmol L^−1^) and generally low TSM from the surface to the bottom. In addition, weak subsurface maxima of BSi and Fuco were obesreved at 50 m at station PN05 (Fig. 4h-j).

In autumn, consistent distribution patterns of salinity, Si(OH)_4_, BSi, and Fuco were distinct throughout the water column at stations PN09 and PN08, demonstrating the highest diatom productivity (BSi of 4.10 µmol L^−1^ and Fuco of 968.7 ng L^−1^ in surface waters) along transect PN mainly driven by replete nutrients in the fresh CCC (Fig. 4k-n). Bottom TSM concentrations (4.5–4.8 mg L^−1^) were clearly high at the two stations but exerted minor influence on BSi and Fuco distributions. Other offshore stations were well mixed charcterized by constant salinites throughout the entire water column. Correspondingly, BSi concentratoins of 0.5–0.6 µmol L^−1^ were comparable between surface and bottom waters at station PN07; those at stations PN05, PN04, and PN03b also varied within a narrow, but lower, range of 0.1–0.2 µmol L^−1^ (Fig. 4k and m). Note that BSi concentrations were slightly higher in nearby bottom waters than in the overlying waters at stations PN05 and PN04, while Si(OH)_4_ peak values of > 10 µmol L^−1^ were observed below 75 m at station PN04 (Fig. 4l, m).

In winter, waters at each station were thoroughly mixed from the surface to the bottom or 100 m indicated by vertically uniform salinity and Si(OH)_4_ concentratoins. Suspended particle distributions, overall reflecting this pattern, were to some extent more dynamic (Fig. 4p-t). High concentrations of BSi (0.5–0.7 µmol L^−1^) throughout the entire water column were observed at stations PN09 and PN08 corresponding to high Fuco (78–98 ng L^−1^) and TSM concentrations (1.5–2.1 mg L^−1^). Notable influence of sediment resuspension may be excluded since these TSM values were lower than maxima observed in PN09 and PN08 bottom waters during other three seasons (Fig. 4e, j, o, and t). Diatom production thus most likely contributed to these abundant particle matters under high Si(OH)_4_ conditions supported by enhanced vertical mixing. In addition, BSi and Fuco concentrations at station PN04 were clearly higher than its neighboring stations PN05 and PN03b, both of which showed weak subsurface maxima of BSi and Fuco around 75 m depth (Fig. 4r, s).

## 4 Discussion

Given the distinct spatial and seasonal variation of BSi in the ECS, diatoms played a changing role in the phytoplankton community and the photosynthetic C fixation, which are examined below by comparing BSi standing stock with that of Fuco, Chl-*a*, and POC (Table 2). Subsequently, environmental factors influencing BSi distributions in the shallow and deep water column are discussed. The BSi standing stock data obtained in this study are further compared with those in the norhtern South China Sea (NSCS), which is the largest marginal sea of the Pacific Ocean.

### 4.1 Biogenic silica vs. fucoxanthin, chlorophyll a, and particulate organic carbon

**Fig. 5:**
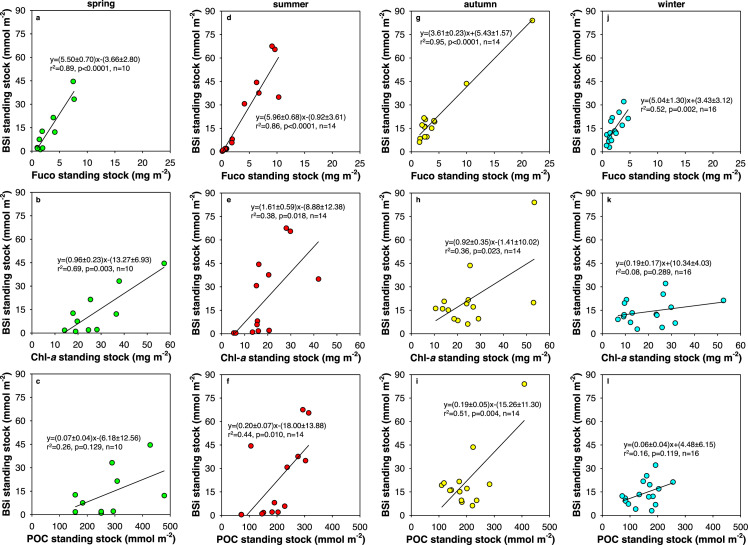
**Biogenic silica (BSi) standing stock vs. that of fucoxanthin (Fuco), chlorophyll *a* (Chl-*a*), and particulate organic carbon (POC) in the shallow water column of the East China Sea in different seasons.** (a-c) spring; (d-f) summer; (g-i) autumn; (j-l) winter. The solid line and the equation in each panel indicate the linear regression fit to the data points.

The standing stock of both BSi and Fuco in the shallow water column was generally higher in summer and autumn (up to 83.9 mmol m^−2^ and 21.9 mg m^−2^, respectively) than in winter and spring (up to 44.5 mmol m^−2^ and 7.6 mg m^−2^, respectively), reflecting high diatom productivity, as well as PP, in warm seasons in the ECS, primarily due to favorable growth conditions such as suitable temperature and sufficient light availability [12,^23^,24]. Strong positive relationships between the standing stock of BSi and that of Fuco were observed in spring, summer, and autumn (coefficient of determination r^2^ = 0.89, 0.86, and 0.95, correlation coefficient significant at *p* < 0.0001, < 0.0001, and < 0.0001, *n* = 10, 14, and 14, respectively), while a moderate positive relationship was observed in winter (r^2^ = 0.52, *p* = 0.002, *n* = 16) (Fig. 5a, d, g, and j). This suggests that diatom-produced opal was the dominant contributor to the observed BSi concentrations in the shallow water column across the entire research domain. The slightly weaker correlation in winter might result from the interference of other phytoplankton containing Fuco, such as chrysophytes [25,26], whose fraction markedly increses in the winter phytoplankton community of the ECS along with notable decline in diatom abundances [12].

Similarly, moderate positive correlations between BSi and Chl-*a* standing stocks were observed in spring, summer, and autumn (r^2^ = 0.69, 0.38, and 0.36, *p* = 0.003, 0.018, and 0.023, *n* = 10, 14, and 14, respectively), whereas data obtained in winter yielded an insignificant correlation (r^2^ = 0.08, *p* = 0.289, *n* = 16) (Fig. 5b, e, h, and k). This difference suggests that diatoms are dominant in the phytoplankton community during the former three seasons in the ECS, but their role is substantially weakened in winter when cryptophytes, prasinophytes, and chrysophytes become more important [12].

Different from the above two analyses, moderate and significant relationships between the standing stock of BSi and that of POC were observed in summer and autumn (r^2^ = 0.44 and 0.51, *p* = 0.010 and 0.004, *n* = 14 and 14, respectively) but insignificant relatiohships in spring and winter (r^2^ = 0.26 and 0.16, *p* = 0.129 and 0.119, *n* = 10 and 16, respectively) (Fig. 5c, f, i, and l), indicating higher C fixation driven by diatoms in warm than in cold seasons in the ECS [12,23]. The slope of linear regressions reflects the bulk particulate Si:C molar ratio and equals 0.20 ± 0.07 and 0.19 ± 0.05 in summer and autumn, respectively (Fig. 5f, i). Both values are within errors consistent with or slightly higher than the average diatom Si:C molar ratio of 0.13 [27], supporting that diatom growth substantially contributed to POC accumulation in the shallow water column during warm seasons. Despite insignificant relatiohships between BSi and POC, the slope values obtained during the two dry seasons (0.07 ± 0.04 and 0.06 ± 0.04; Fig. 5c, l) were lower than 0.13. In this case, diatoms’ contribution to POC standing stock was exceeded by other phytoplankton such as dinoflagellates in spring and cryptophytes in winter [11,12]. Consequently, BSi vs. POC relationships in the shallow water column varied in different seasons in the ECS, which were mainly associated with changes in phytoplankton community structure.

### 4.2 Factors influencing biogenic silica distributions

**Fig. 6:**
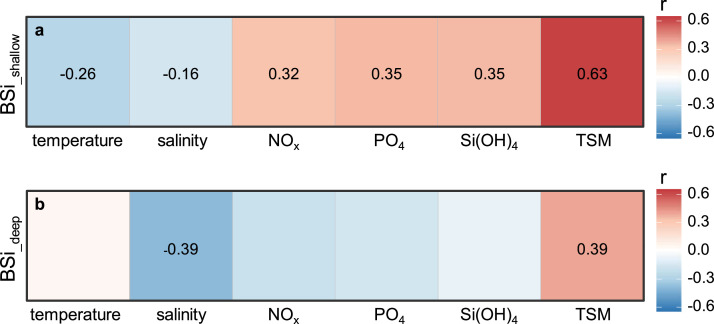
**Relationships between biogenic silica (BSi) and various environmental factors including temperautre, salinity, nutrients (nitrate+nitrite (NO_x_), phosphate (PO_4_), and silicate (Si(OH)_4_)), and total suspended matter (TSM).** (a) Above the integrating depth of BSi standing stock; (b) below the integrating depth of BSi standing stock. The numbers in boxes denote the coefficient of determination (r) significant at *p* < 0.05; boxes without numbers denote insignificant correlations.

Using data collected during all four seasons, we analyzed relationships between BSi and various environmental factors in the ECS shallow and deep water columns, respectively, which are divided by the integrating depth of BSi standing stock at each station (Fig. 6; Table 3).

**Table 3:** **Values of coefficient of determination (r) and correlation coefficient significance (p) for relationships between biogenic silica (BSi) and various environmental factors including temperautre, salinity, nutrients (nitrate+nitrite (NO**_**x**_**), phosphate (PO**_**4**_**), and silicate (Si(OH)**_**4**_**)), and total suspended matter (TSM)**.

		temperature	salinity	NO_x_	PO_4_	Si(OH)_4_	TSM
BSi_-shallow_^a^	r	−0.26	−0.16	0.32	0.35	0.35	0.63
	p	0.0008	0.0473	3.93E-5	7.14E-6	8.18E-6	7.32E-19
BSi_-deep_^b^	r	0.05	−0.39	−0.19	−0.16	−0.08	0.39
	p	0.7092	0.0008	0.1090	0.1839	0.5254	0.0009

a Above the integrating depth of BSi standing stock.

b Below the integrating depth of BSi standing stock.

In the shallow water column, BSi showed the strongest postive relationship with TSM mainly composed of biogenic particles. This further suggests diatom production largely controlled BSi distributions, which is supported by moderate positive correlations between BSi and nutrients. On the other hand, BSi showed weak, but significant, negative relationships with both temperature and salinity (Fig. 6a; Table 3). This mainly results from the fact that the cold and fresh CCC, as well as river discharges including the CDW, have generally high BSi concentrations since these water masses are enriched in nutritens stimulating high diatom productivity.

In the deep water column, BSi showed a moderate postive relationship with TSM, which is notably influened by sediment resuspension at nearshore stations. Benthic inputs may have thus contributed to some extent to BSi enrichment in nearby bottom waters. On the other hand, BSi showed a moderate negative relationship with salinity, reflecting overall negligible diatom prodcution in the saline and dark waters despite under high-nutrient conditions. This essentially leads to insignificant correlations observed between BSi and NO_x_, PO_4_, and Si(OH)_4_ in deep waters of the ECS (Fig. 6b; Table 3).

### 4.3 Comparison with the South China Sea

The ECS and the SCS are two major marginal sea systems of the wNP, which are connected by water mass exchange through the Taiwan Strait [28] and offshore through the KC [29]. The ECS is dominated by a broad and relatively eutrophic shelf, whereas the SCS is characterized by an oligotrophic and permanently stratified deep basin. The contrasting physical and/or chemical conditions between the two ocean margins imply various roles of diatoms in phytoplankton community and C fixation. While differences in seawater Si dynamics have been briefly discussed associated with Si(OH)_4_ distributions [28] and stable Si isotopes [13], we systematically compare BSi in the shallow water column, defined as BSi contents approximating BSi standing stock/integrating depth, of the ECS with those of the NSCS during the same four seasons [7].

**Fig. 7:**
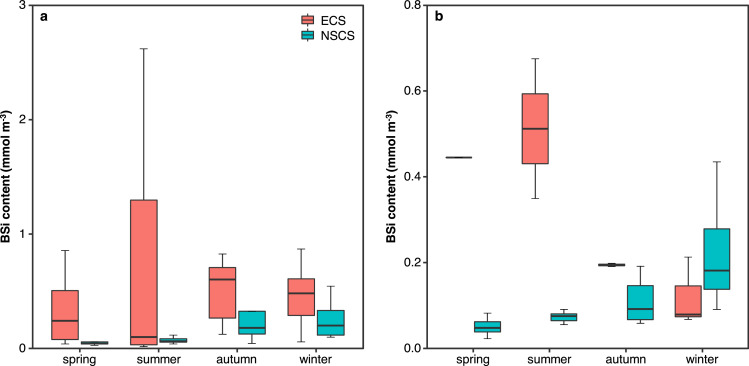
**Comparsions of biogenic silica (BSi) contents (approximating BSi standing stock/integrating depth) in the shallow water column between the East China Sea (ECS) and the northern South China Sea (NSCS) in different seasons.** (a) For stations shallower than 200 m; (b) for stations deeper than 200 m.

On the shelf with water depth < 200 m, BSi contents in the ECS were overall higher than or within errors comparable to those in the NSCS (Fig. 7a). During each season on the ECS shelf, BSi contents varied within a generally wide range reflecting large spatial variability, which results in inapparent seasonal variabilty. On the NSCS shelf, however, BSi contents were clearly higher in autumn and winter than in spring and summer, indicating higher diatom productivitiy in the dry than wet season [7,30]. The NSCS shelf is fed by the Pearl River plume in the wet season and enhanced vertical mixing, as well as the CCC flowing across the Taiwan Strait from the ECS, in the dry season, all of which bring nutrients favoring diatom growth. This to some extent explains no significant differences in BSi contents were observed between the two systems, in particular in autumn and winter (Fig. 7a). Note that prior studies have demonstrated that summer PP is generally higher than in other seasons on the ECS shelf [12,^23^,24] and PP displays minor differences between seasons on the NSCS shelf [31], [32], [33]. The former is supported by the extremely high summer maximum of BSi contents, despite the seasonal variability on the ECS shelf was insignificant in statistics (Fig. 7a). For the latter, the temporal variations in diatom productivitiy may have been too small to affect the seasonal pattern of PP, which is also contributed by the nano- and pico-phytoplankton [31,34].

In the slope/basin with water depth > 200 m, BSi contents in the ECS were notably higher than those in the NSCS in spring and summer (Fig. 7b), reflecting different trophic levels of the two systems. The ECS slope is generally eutrophic with nutrient supplies from the upwelled Kuroshio subsurface water, whereas the NSCS slope and basin are overall oligotrophic because strong stratification inhibits upward transport of nutrients from deep waters. Similar to the shelf scenario, BSi contents in the NSCS slope and basin increased in autumn and winter, which indicates enhanced diatom productivity [7,30], as well as PP [33], stimulated by high nutrient supplies through vertical mixing. In contrast to the shelf scenario, however, BSi contents in the ECS slope drastically decreased in autumn and winter mainly due to unfavorable growth conditions for diatoms such as low temperature and reduced light availability [12,^23^,24]. As a consequence, BSi contents were within errors comparable between the two systems in the dry season (Fig. 7b). Nevertheless, diatoms play a more important role in the phytoplankton community in the slope/basin of the ECS [11,12] than of the NSCS dominated by the picophytoplankton [31,34]. Diatoms in the former are thus more sensitive to the markedly changing physical and/or chemical conditions resulting in the larger seasonal variability of BSi contents.

## 5 Concluding remarks

Water column BSi concentrations showed notable spatial and seasonal variability in the ECS, which generally decreased from the shelf to the slope and were overall higher in summer and autumn than in winter and spring. Varying correlations between the standing stock of BSi and that of Fuco, Chl-*a*, and POC in the shallow water column pointed to changing diatoms’ role in the phytoplankton community and the photosynthetic C fixation. Diatoms were ecologically more important in spring, summer, and autumn than in winter, while the POC accumulation was substantially contributed by diatoms during summer and autumn. Signifcant positive relatioships between BSi and nutrients and TSM were observed in the shallow water column based on all samples and data collected in the ECS. This reflects that high diatom productivity, stimulated by rich nutrient supplies mainly from river plumes (e.g., the CDW), the CCC, and/or the KBC, represents a large fraction of biogenic particles.

It is possible that current BSi dynamics in the ECS differ from those indicated by our observations conducted more than a decade ago, which warrants further investigation by collecting new samples and data of water column BSi. Nevertheless, we suggest that despite local changes that may have occurred (e.g., increased nonsiliceous algae over diatoms off the Changjiang Estuary [35]), the overall distribution pattern of BSi in the ECS presented in this study should stand, particularly given that no significant changing trend of Si(OH)_4_ fluxes was observed near the Changjang river mouth [36,37]. Relative to extensive work regarding BSi in the ECS sediments, our data and results suggest that high-resolution sampling of biogenic particles throughout the entire water column is required for a comprehensive understanding of Si cycling, including BSi production, settlement, and bury, and its inteaction with C cycling in a eutrophic marginal sea.

## Declaration of competing interest

The authors declare that they have no conflicts of interest in this work.
